# Reduced postoperative pain in patients receiving nociception monitor guided analgesia during elective major abdominal surgery: a randomized, controlled trial

**DOI:** 10.1007/s10877-022-00906-1

**Published:** 2022-08-17

**Authors:** Rivka Fuica, Carlos Krochek, Rachel Weissbrod, Dimitri Greenman, Andres Freundlich, Yaacov Gozal

**Affiliations:** 1 Department of Anesthesiology, Perioperative Medicine and Pain Treatment, Shaare Zedek Medical Center, PO Box 3235, Jerusalem, Israel; 2 Medasense Biometrics Ltd, Ramat Gan, Israel

**Keywords:** Nociception, Nociception level-guided analgesia, Opioid, Postoperative pain, Artificial Intelligence, Personalized Anesthesia

## Abstract

The Nociception Level index (NOL™) is a multiparameter index, based on artificial intelligence for the monitoring of nociception during anesthesia. We studied the influence of NOL-guided analgesia on postoperative pain scores in patients undergoing major abdominal surgery during sevoflurane/fentanyl anesthesia. This study was designed as a single-center, prospective randomized, controlled study. After Institutional Review Board approval and written informed consent, 75 ASA 1–3 adult patients undergoing major abdominal surgery, were randomized to NOL-guided fentanyl dosing (NOL) or standard care (SOC) and completed the study. The sevoflurane target MAC range was 0.8–1.2. In the NOL-guided group (N = 36), when NOL values were > 25 for at least 1 min, a weight adjusted fentanyl bolus was administered. In the control group (N = 39) fentanyl administration was based on hemodynamic indices and clinician judgement. After surgery, pain, was evaluated using the Numerical Rating Scale (NRS) pain scale, ranging from 0 to 10, at 15 min intervals for 180 min or until patient discharge from the PACU. Median postoperative pain scores reported were 3.0 [interquartile range 0.0–5.0] and 5.0 [3.0–6.0] at 90 min in NOL-guided and control groups respectively (Bootstrap corrected actual difference 1.5, 95% confidence interval 0.4–2.6). There was no difference in postoperative morphine consumption or intraoperative fentanyl consumption. Postoperative pain scores were significantly improved in nociception level index-guided patients. We attribute this to more objective fentanyl dosing when timed to actual nociceptive stimuli during anesthesia, contributing to lower levels of sympathetic activation and surgical stress. Clinicaltrials.gov identifier: NCT03970291 date of registration May 31, 2019.

**Supplementary Information:**

The online version contains supplementary material available at 10.1007/s10877-022-00906-1.

## Introduction

The administration of analgesic drugs such as opioids during general anesthesia is determined by interpreting vital signs in the context of the clinical experience of the anesthesiologist [1]. Sufficient analgesia during surgery is critical to avoid hypertension, tachycardia, vasoconstriction and other sympathetic nervous system responses, as well as unexpected patient movement and pain sensitization. However, opioid overdosing may cause major and minor side effects, including postoperative respiratory depression, nausea and vomiting, ileus, pruritis, opioid-induced hyperalgesia, and others [2].

Opioid drugs are titrated based on clinical signs of stress-induced activation of the sympathetic system such as an increase in heart rate, blood pressure, lacrimation, and sweating [3]. However, changes in these physiological measures may be affected by the administration of beta-blockers, neuromuscular blocking agents, anticholinergics, and opioids, which cause vasodilation, paralysis, mydriasis, and myosis. Hence, their interpretation is highly subjective.

Although recent research has identified some variables that predict severe postoperative pain and opioid consumption [4], limited data are available concerning the intricate relationship between intraoperative analgesic management and pain on arrival in the post-anesthetic care unit (PACU).

A continuous, objective nociception monitor may reduce the subjectivity in dosing analgesics, reduce the risk of overdosing or underdosing, and improve patient safety during general anesthesia. Poorly controlled acute postoperative pain is associated with increased morbidity, functional and quality-of-life impairment, delayed recovery time, and higher health-care costs [5].

**Fig. 1:**
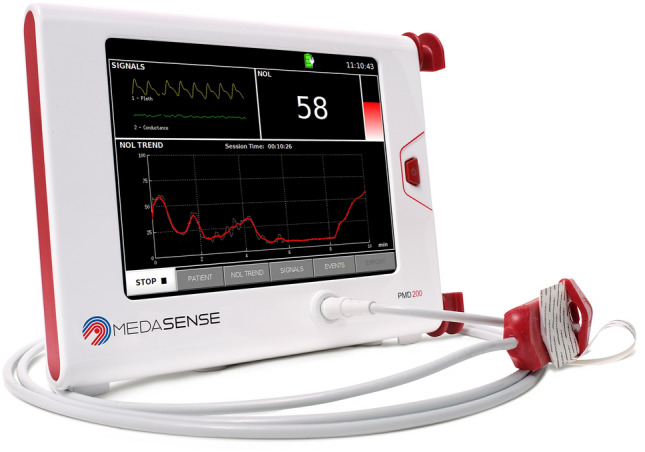
The PMD-200 Monitor

The PMD-200 monitor (Medasense Biometrics Ltd, Ramat Gan, Israel) makes use of an algorithm based on advanced machine learning technologies; it combines photoplethysmogram (PPG) amplitude, skin conductance, heart rate, heart rate variability and their time derivatives into a single index, the NOL-index [6]. Machine learning was used to create the optimal algorithm to translate input (predictors) into output (NOL-index) without the need of an a priori specified stochastic model. The index ranges from 0 (absence of nociception) to 100 (extreme nociception) (Fig. 1). The algorithm was validated in multiple studies [7, 8] with a NOL value of 25 identified by the manufacturer as the `best fit` cut-off score to discriminate between nociceptive and non-nociceptive response [9]. The algorithm furthermore ‘personalizes’ its nociception reading to the individual patient by ‘learning’ the magnitude of the physiologic responses to surgical stimuli as the case progresses and calibrating its output accordingly. The performance of the monitor in patients treated with chronic beta blockers was validated by Bergeron et al. [10].

Recently, Meijer and colleagues reported that NOL guided fentanyl-administration during sevoflurane/fentanyl anesthesia in major abdominal surgery results in a reduction of 1.6-point in Numerical Rating Scale (NRS) pain scores in the NOL-guided group in the post anesthesia care unit [11]. Since EEG monitoring is not a standard of care at our institution, we used MAC target to ensure adequate hypnosis during general anesthesia.

We hypothesized that intraoperative NOL-guided fentanyl administration reduces post-operative pain scores after elective major abdominal surgery The primary objective of this prospective, controlled trial was to assess the clinical effect of NOL guided fentanyl dosing on post-operative pain. The secondary objectives of this study were to evaluate the effects of NOL monitoring on the frequency of inadequate anesthesia events and post operative analgesia requirement in the PACU.

## Materials and methods

This single center, prospective, single blinded two-arm, parallel, randomized controlled superiority study was approved by the hospital’s Ethics Committee and the study protocol was published on clinicaltrials.gov (NCT03970291) on May 31, 2019 before patient enrollment began. The study was conducted at Shaare Zedek Medical Center, Jerusalem, Israel from November 1, 2019 through May 31, 2021. All patients were approached by the principal investigator, and after presentation of the study purposes, written informed consent was obtained. Minor protocol amendments were made to include a sample size rationale, extend recruitment period, remove an exploratory endpoint which could not be measured and reword an exclusion criterion to improve patient enrollment rate. Five anesthesiologists performed the study cases.

### Inclusion and exclusion criteria

Adult patients, ASA PS I-III (American Society of Anesthesiology Physical Status Class) scheduled for major elective laparoscopic abdominal, urologic or gynecologic procedures under general anesthesia without a planned epidural or regional block were eligible for inclusion.

Exclusion criteria were pregnancy, non-sinus heart rate, severe cardiac arrhythmias, central nervous system disorder, alcohol or illicit drugs abuse within the last six months, chronic pain conditions, opioid tolerance, chronic use of psychoactive drugs and surgery duration of less than one hour.

Subjects could withdraw from the study at any time without prejudice. The investigator could withdraw a subject from the study if deemed to be in the best interest of the subject or if the subject could not comply with elements of the protocol that were critical for safety or necessary for the scientific integrity of the study. Subjects enrolled in the study, who the researchers decided for any reason should not continue in the study (“dropouts”), were replaced.

### Randomization and blinding

Randomization to either NOL-guided analgesia or standard care was performed using the electronic data capture system CASTOR (https://www.castoredc.com) in the operating room prior to induction of anesthesia. Patients, surgeons, and PACU nurses were not informed of the group assignment. In both allocation groups, the NOL monitor (PMD-200, Medasense Biometrics Ltd., Ramat Gan, Israel) was connected to the patient by finger probe, placed on the left or right middle finger. In case of NOL-guided analgesia, the monitor screen was visible to the anaesthesia team and used to guide fentanyl administration. In case of standard care, the clinician was blinded to the nociception monitor but the NOL index was recorded by the monitor.

### Anesthesia procedure

#### Perioperative clinical care

Patients did not receive sedatives or pre-emptive analgesics prior to the induction of anesthesia. Patients from both groups received an induction dose of propofol (2mg/kg), fentanyl (1-2 µg/kg) and rocuronium (0.6 mg/kg). Standard anesthesia monitors were used and a target MAC of sevoflurane 0.8–1.2 was obtained post induction.

During emergence, residual neuromuscular block (train-of-four ratios <0.9) was reversed with sugammadex 2 mg/kg and patients were extubated when neuromuscular function had normalized (train-of-four ratio > 0.9), were breathing spontaneously, and responded to commands. Each subject received IV acetaminophen 1g, IV morphine (0.1– 0.15 mg/kg) and IV ondansetron 4mg, 30–45 min before the end of surgery.

In the PACU, additional intravenous doses of morphine or tramadol were given according to standard PACU clinical guidelines.

### Treatment in the maintenance phase

#### Fentanyl administration in the NOL-guided group

For the NOL-guided group patients, the administration of intraoperative fentanyl IV was guided by 60 sec trends of the NOL-index. In cases where the NOL index was above 25 for at least 60 sec, a bolus of 0.5 µg/kg fentanyl was administered and repeated every 5 min until NOL-index scores decreased below 25. This bolus regimen was conservative as a precaution but consistent with clinical practice at the institution. MAP (Mean Arterial Pressure) and HR (Heart Rate) were monitored and always considered.

When the NOL-index decreased below 25, no more fentanyl was administered. If the NOL-index was below 25 and the MAP below 60mmHg, vasoactive medication (ephedrine, phenylephrine, norepinephrine), crystalloids, or both could have been given.

#### Fentanyl administration in the standard care group

For the control group patients, fentanyl IV was dosed according to the clinician’s clinical judgement.

When MAP was > 100 mmHg, a vasodilator or a bolus of fentanyl could be given, and for hypotension—(MAP < 60 mmHg), the sevoflurane concentration was lowered, and vasoactive medications and/or fluids were administered, according to the judgement of the clinician.

### Patient management in the PACU

Pain scores were measured upon arrival in the PACU and every 15 minutes until discharge or up to a stay of 180 minutes by a trained research assistant using the NRS pain score. Doses of morphine or tramadol IV to treat pain were given according to standard PACU clinical guidelines at our hospital.

Nursing staff recorded the incidence of nausea, vomiting and the requirement for antiemetic medication.

Patients were ready for discharge when the Modified Aldrete Score recorded by nursing staff reached 9, and pain score (NRS) was below 4.

### Post-Surgery Follow-Up

Pain scores recorded up to 24 hours from the end of surgery were collected according to the standard of care, typically twice a day. Information regarding morphine consumption, nausea, vomiting and rescue medications was collected.

## Main outcome measures

The primary outcome measure was the pain score in the PACU measured at arrival, every 15 min and at discharge or at 3 h whichever came first. Our secondary outcome measures were the frequency of inadequate anesthesia events during the maintenance period until reversal defined as: MAP < 55 mmHg (severe hypotension), MAP < 60 mmHg (hypotension); SBP (Systolic Blood Pressure) greater than 140 mmHg; HR less than 45/min; HR greater than 90/min and total intraoperative fentanyl consumption (in µg). Other hypothesis-generating outcome measures included:

1. Frequency of vasoactive medication (ephedrine, phenylephrine, norepinephrine, atropine) intraoperatively and in the PACU.
2. Time to the first administration of morphine and/or non-opioid systematically administered analgesics;
3. Post-operative opioid consumption from arrival to discharge;
4. Readiness to discharge from PACU;
5. Post-operative sedation scores with Ramsay Sedation Score
6. Respiratory Depression- in PACU as defined as respiratory rate (RR) below 8 respirations per minute (RPM) for 1 min, oxygen saturation of less than 90% for 1 min under continuous monitoring.
7. Nausea and vomiting incidence (PONV Score)
8. Pruritis requiring treatment.

### Data collection

Data was collected using the CASTOR eCRF with medical records, score sheets of study measures, and source documents as the primary source of data. Monitor recordings were downloaded for subsequent analysis by the sponsor. All data was pseudonymized and validated prior to database lock and analysis.

### Study power

In order to demonstrate a clinically meaningful reduction in PACU pain scores of two points [11] in the NOL guided group with a one-sided alpha level of 5.0%, a power of 80%, and a dropout rate of 10%, we planned on enrolling 84 subjects.

### Statistical analysis

For the analysis of continuous variables, mean, median and standard deviations were calculated. Box plots were used to present the changes in NRS pain scores. Because each patient had multiple pain evaluations and these measures were not independent, an analysis of repeated measures was performed using a bootstrapping analysis.

Statistical significance was set at p < 0.05 and 95% confidence intervals were reported. No interim analysis was planned.

For secondary outcomes of interest, continuous variables were compared between the two groups using a Student t-test for normally distributed variables or Mann–Whitney U-test for non-normally distributed variables. Analysis of inadequate anesthesia events was performed at 5-min intervals. Analyses were performed using the R Stats package.

## Results

### Patient population

**Fig. 2:**
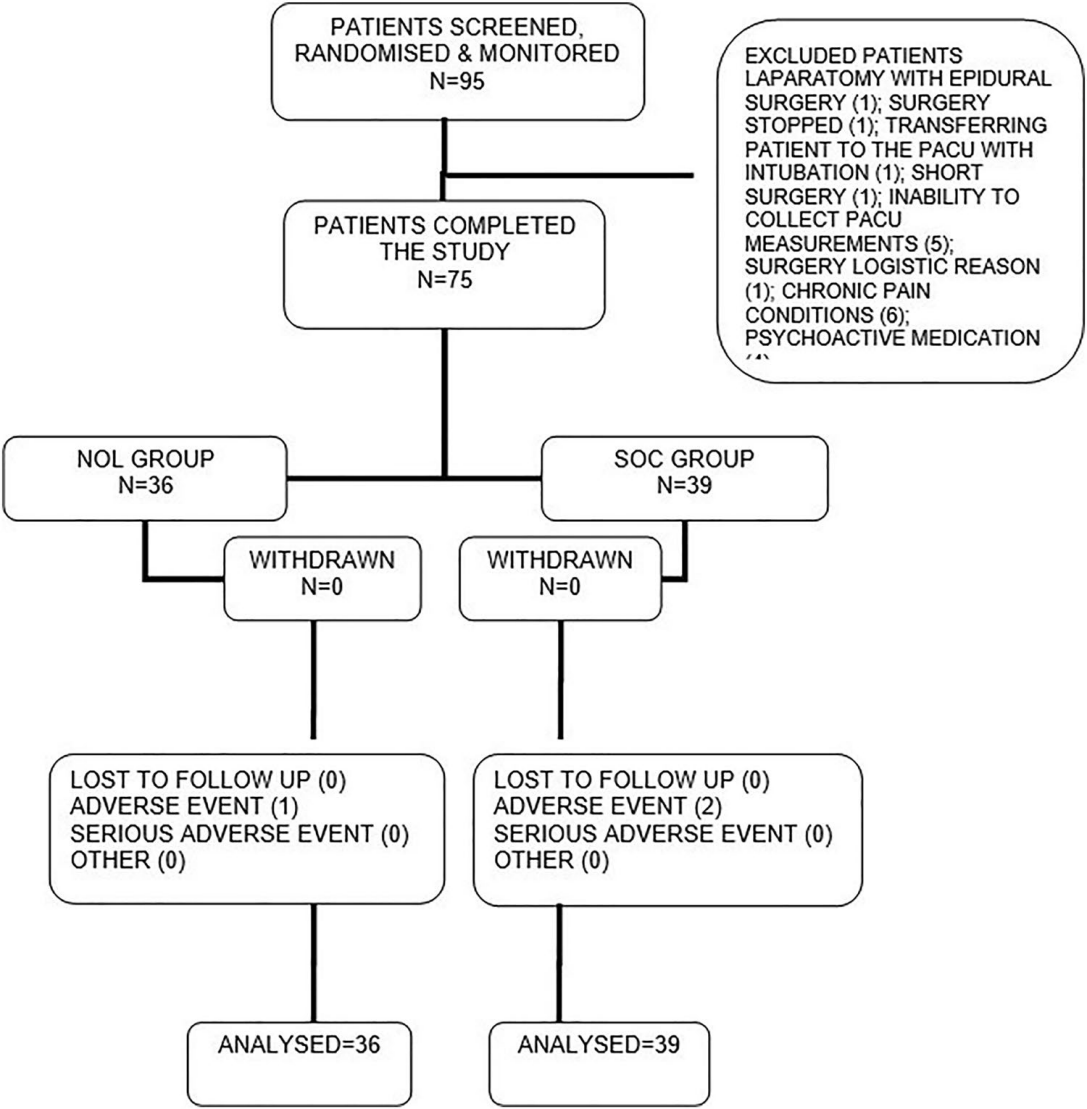
Patient flow chart

Between November 2019 and April 2021, A total of 95 patients were approached for participation in the study. Ten patients who were enrolled and randomized, were excluded post factum since they reported suffering from chronic pain or using psychoactive medication (which was a protocol exclusion criteria). These patients were withdrawn from the study after completing the study procedures but prior to statistical analysis. Ten other patients were randomized but did not complete study procedures for various reasons: conversion of laparoscopy without epidural to laparotomy surgery with epidural (1); Transferring an intubated patient to PACU who could not report pain scores (1); Surgery stopped due to respiratory distress associated with Trendelenburg position (1); Short surgery (1); Inability to collect PACU measurements (5); Surgery logistic reason (1). Seventy-five (75) patients completed the study with no major protocol deviations with 36 patients in the NOL guided group and 39 randomized to the control group. The patient flow diagram is shown in Fig. 2.

Patient Demographics are reported in Table 1. There were no statistically significant differences between the groups. Variables collected during and after surgery were similar in both treatment groups as summarized in Table 2, with the exception of PACU pain scores which differed. All values are represented as mean or actual differences (95% confidence interval).

**Table 1:** Patient Demographics Summary

	NOL (N = 36)	Control (N = 39)	p value
Age, Mean (CI)	60 (55–65)	57 (51–6)	0.43
Sex			0.07
Male, n (%)	12 (33%)	21 (54%)	
Female, n (%)	24 (67%)	18 (46%)	
BMI, Mean (CI)	27 (25–30)	27 (26–29)	0.58
ASA			0.60
1, n (%)	4 (11%)	7 (18%)	
2, n (%)	25 (69%)	23 (59%)	
3, n (%)	7 (20%)	9 (23%)	
Type of surgery			0.09
Urology (prostatectomy, cystectomy, pyeloplasty)	7 (20%)	15 (38%)	
Gynecology (hysterectomy, myomectomy)	13 (36%)	15 (38%)	
General Surgery (colectomy, nephrectomy, appendectomy, bariatric)	16 (44%)	9 (24%)	

All values are represented as mean ± 95% *CI* or numbers n (%), *BMI* body mass index

**Table 2:** Variables collected during and after surgery

Variable	NOL Group (N = 36)	SOC (N = 39)	Mean or actual difference (95% CI)	P-value
Anesthesia duration (min)	190	207		0.50
Surgery time (min)	227	238		0.61
End-tidal sevoflurane conc. (%)	2	2		0.47
Total fentanyl consumption for the entire surgery (µg))	291	273	18 [−46to 82]	0.61
Average fentanyl consumption per hour (µg.h^−1^)	107 (54)	101 (52)		0.62
Average time of last fentanyl bolus from end of surgery (min)	93 (58)	119 (73)		0.12
Reversal time (min)	11	11	0.32 [−4.46 to 3.82]	0.67
Morphine & morphine equivalents consumed in PACU (mg/kg)	0.12	0.11	0.01 [−0.04 to 0.05]	0.55
Aldrete score at discharge	9.3	9.4	0.09 [−0.39 to 0.21]	0.67
Patients with Aldrete score > 8 at PACU discharge (%) and breakdown by score	92 (8–8%,9–47%, 10–45%)	100 (9–72%, 10–28%)		
Time spent in the PACU (min)	161	143	17.46 [−16.19 to 51.71]	0.13
HR baseline (bpm)	77	80	3.68 [−9.71 to 2.34]	0.18
MAP baseline (mmHg)	95	98	2.58 [−8.14 to 2.98]	0.43
Inadequate Anesthesia Events (normalized to surgery duration event/h)	NOL Group (N = 36)	SOC (N = 38)		
HR > 90 (bpm)	0.13	0.13	0.00 (−0.19 to 0.18)	0.65
HR < 45 (bpm)	0.06	0.03	−0.02 (−0.09 to 0.05)	0.84
SBP > 140 (mmHg)	0.63	0.51	−0.11 (−0.44 to 0.21)	0.71
MAP < 60 (mmHg)	0.62	0.37	−0.25 (−0.52 to 0.03	0.14
MAP < 55 (mmHg)	0.33	0.10	−0.23 (−0.42 to −0.04)	0.07

## Outcomes

### Primary endpoint

Patient pain scores were collected every 15 min and a median pain score was calculated for each patient using generalized linear models with the cluster bootstrap and bias corrected and accelerated (Bca) 95% confidence intervals (CI).

**Fig. 3:**
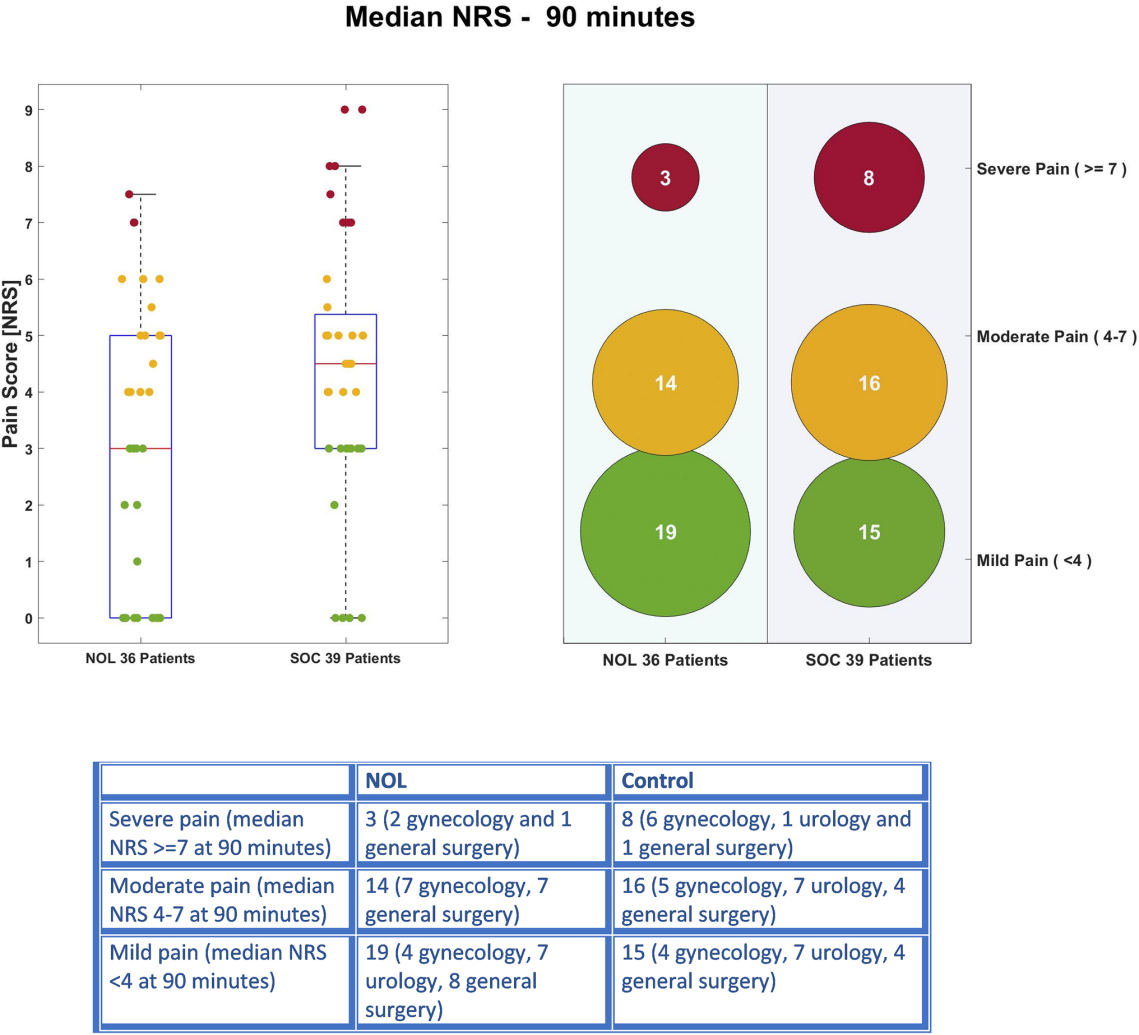
PACU pain Scores at 90 minutes with breakdown by surgery type

PACU pain scores for the first 90 min in the PACU are presented in Fig. 3. Pain scores were consistently higher in patients that had received standard care compared to those that had received fentanyl dosing guided by the NOL index. The patients median pain scores in the PACU were 3.0 [0.0–5.0] and 5.0 [3.0–6.0] in NOL guided and control groups, respectively (Bootstrap actual difference 1.3 with 95% confidence interval 0.3 to 2.3).

As there were notable differences in the distribution of males and females between groups (33.3% & 66.7% respectively in the NOL group vs. 53.8% & 46.2% in the SOC group, p = 0.07), the Bootstrap model was corrected for these differences. Results remained statistically significant with a corrected Bootstrap model providing an actual difference of 1.5 with 95% confidence interval 0.4 to 2.6.

The maximal pain score in the NOL group over 90 min in the PACU was 4.8 in the NOL group vs. 6.6 in the SOC group, actual difference 1.8, p = 0.006.

In order to demonstrate applicability to US perioperative practices, the pain score results were calculated also for 60 min in the PACU, since that is the typical length of stay after major surgery in the US. The patients median pain scores in the PACU were 3.0 [0.0–5.0] and 5.0 [3.0–6.0] in NOL and control groups, respectively (Bootstrap actual difference 1.6 with 95% confidence interval 0.7 to 2.7). Results remained statistically significant with a Bootstrap model corrected for differences in sex distribution providing an actual difference of 1.9 with 95% confidence interval 0.7 to 3.0.

**Fig. 4:**
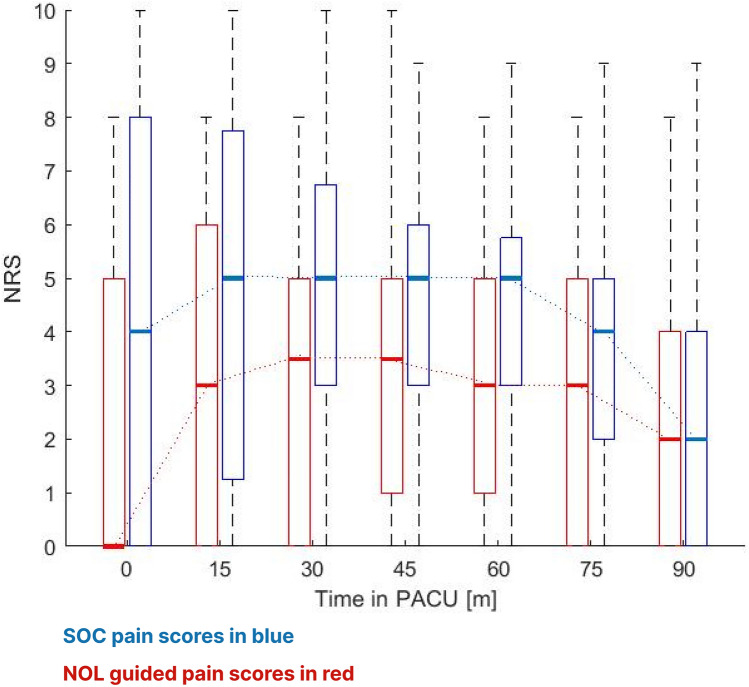
PACU pain score trajectories in the NOL guided and SOC groups

The bubble diagram in Fig. 3 demonstrates that in the NOL guided group the number of patients reporting mild pain was higher in the NOL group than in the control group, whereas the number of patients reporting severe pain was lower in the NOL group even when adjusting for the difference in number of subjects between the two groups. The distribution of the different surgery types is provided showing that most of the patients reporting severe pain in both groups underwent gynecological procedures. It is also of interest to note that in the NOL guided group, all the patients undergoing urological surgery reported mild pain. As the study was not designed to detect differences in particular surgery types, these results should be considered hypothesis generating only. Figure 4 describes the 90 min median pain score trajectories of the NOL guided and the SOC groups demonstrating the lower pain scores in the NOL group throughout the PACU stay.

### Secondary & exploratory endpoints

There were no differences in the prevalence of inadequate analgesia/anesthesia events between the NOL and the SOC reported in Table 2. Hemodynamic data for one of the patients in the SOC group were lost due a technical issue. Due to differences in surgery length, all results were normalized by surgery time and reported as mean number of events per hour. The patients were monitored with an arterial line or non-invasive blood pressure according to the decision of the clinician. Hemodynamic data was analyzed at intervals of 5 min. The difference in severe hypotension events (MAP < 55 mmHg) was borderline statistically significant with 0.33 events/h in the NOL group and 0.10 events/h in the SOC group, p 0.07 absolute difference −0.23 (−0.42 to −0.04 CI). However, this finding is not considered clinically meaningful. There was no difference in the mean number of events per patient treated with vasoactive drugs in both groups which further supports the lack of clinical significance (2.8 treated events in the NOL group and 2.8 events in the SOC group p = 0.88).

There were no differences in total intraoperative fentanyl consumption between the NOL and the SOC with a mean dose of 291 µg in the NOL group and 273 µg in the SOC group (Mann–Whitney p = 0.61). No difference in fentanyl consumption per hour were found when normalizing for surgery duration (107 µg.h^−1^ in the NOL group and 101 in the SOC group p = 0.62) suggesting that surgery duration did not create a confounder. In order to assess whether the timing of fentanyl dosing may have impacted pain scores we compared the mean timing of the last fentanyl bolus from the end of surgery between the two groups. There was no significant difference with the last bolus given 93 min before the end of surgery on average in the NOL group and 119 min before surgery end in the SOC group (p = 0.64).

There were no significant differences reported in the time spent in PACU and in post operative opioid consumption or in any of the other exploratory endpoints. No device related adverse events were reported during the study.

### Post hoc analysis

As we did not find a difference in fentanyl dosing, we performed a post-hoc unplanned analysis to assess differences in the NOL values in the two study groups that may explain the difference in PACU pain scores. There were no differences in the mean, median or last NOL values measured. Per the protocol guidance, an event of NOL > 25 for at least 60 s may represent a nociceptive event that could be treated with a fentanyl bolus in the NOL guided group. We compared the number of NOL events that could have warranted treatment in both groups (in the control arm, the clinician was blinded to NOL) normalizing for both number of surgeries and surgery duration. The number of events was lower in the NOL guided arm in all analysis forms: The total number of events was 117 in the NOL group and 227 in the SOC group. The average number of NOL events per hour surgery was 1.57 in the NOL group and 1.96 in the SOC group (p = 0.3).

Although this analysis does not demonstrate a statistically significant difference, the approach seems promising and warrants further attention in future studies.

## Discussion

Acute postoperative pain is a common concern of patients undergoing surgery [5]. According to the Institute of Medicine, up to 80% will experience severe pain in the recovery period, and subsequently be at higher risk of developing chronic pain [12].

Sub-optimal acute-pain management in surgery patients is accompanied by an array of negative consequences, including increased morbidity, impaired physical function and quality of life, slowed recovery, prolonged opioid use during and after hospitalization, and increased cost of care. In addition, early postoperative pain appears to trigger persistent pain that may last for months after surgery in a substantial proportion of patients [5]. In addition, high pain scores correlate with low patient satisfaction scores, which are now a quality metric that adversely affects hospital certification, reimbursement, and provider recredentialing.

Our study demonstrates that that NOL-guided fentanyl dosing results in a clinically meaningful and statistically significant reduction in postoperative pain scores when compared to dosing based on hemodynamic indices (BP and HR) and clinical judgement, without adversely impacting patient safety. We intentionally excluded patients with planned regional anesthesia from the study in order to avoid study confounders related to regional anesthesia efficacy that would not be directly affected by NOL monitoring.

At 90 min post operatively which is the typical duration of PACU stay at our hospital, the median reduction was 1.3 in the NOL-guided group. When corrected for the predominance of female subjects in the NOL guided cohort, the differences in pain scores were more pronounced at 1.5 at 90 min respectively. Although we did not show a reduction of 2 points on the NRS scale as hypothesized, the results do meet currently accepted definitions for clinical meaningfulness as demonstrated in recent studies that were powered for an improvement of 1.3 points or an improvement of 30% [13] [14].

The significant difference in the mean and maximal pain scores between the two groups over 90 min further supports the clinical meaningfulness of the results. A clinically meaningful reduction in pain scores may increase patient satisfaction, improve patient outcomes and reduce postoperative opioid consumption.

In many PACUs, the decision to treat pain with opioids and the size of the dose administered is simplified into a three-tiered, pain category (mild, moderate, severe). A consequence of the 1.5 point median decrease in pain scores in the NOL-guided group is that a significant percentage of patients who may have experienced ‘severe’ or ‘moderate’ pain will now experience lower levels of pain, which may reduce the dose of opioids administered in the PACU, and accordingly, the incidence of opioid related side effects expediting PACU discharge.

Dahan et al. present a detailed description of the effect of inter-patient variability on opioid dosing and the possible contribution of a pharmacogenetic effect. After a standard dose of opioid, the inter-patient variability in plasma concentrations is large (at least 30-fold) and related to various factors including weight-related parameters (lean and fat body mass), organ function (hepatic and renal function), and cardiac output [15]. The authors note that the safest approach to opioid analgesia is one of careful titration to analgesic effect during surgery and in the postoperative period, with acute awareness of the undesirable dose-related side effects. An objective, quantitative monitor of the nociception anti-nociception balance can help the clinician reduce the uncertainty of drug effect. In similarity to other non-invasive patient monitors such as pulse oximeters, patient outcomes may be improved by the use of advanced monitors only if the displayed data prompts changes in interventions by the attending clinicians, therefore the importance of clinical implementation in a standardized manner based on clear guidance for interventions is of the utmost importance.

A recently published controlled study in female patients undergoing laparoscopic gynecology procedures reported a significant reduction of 25% in fentanyl concentration normalized to surgery duration in the NOL guided group with no difference in total fentanyl or in the post-operative pain levels reported in both groups [16]. In this study the pain scores were low (averaging less than 3 in both groups) which is attributed to the type of type of gynecological laparoscopic surgery with no large incision on the abdominal wall. The major abdominal surgery procedures included in our study required larger doses of intraoperative fentanyl and were associated with higher pain scores. Other controlled studies have reported reduction in sufentanil and remifentanil consumption with NOL guided analgesia with no effect on pain scores [17] [18] [19]. However, these studies are of less relevance due to the different anesthetic regimen chosen.

In our study, the results of the secondary endpoints indicate that the improvement in PACU pain score in the NOL guided group is not attributed to increased fentanyl consumption both overall and when normalized to surgery duration (per hour), and NOL guided analgesia did not adversely affect hemodynamic stability. The timing of the last fentanyl dose during surgery was not different in the two groups and it is important to note that all patients received pre-emptive morphine 40 min before the end of surgery which would have come into effect during PACU stay in addition to any remaining fentanyl. There was no difference in sevoflurane concentrations between the groups that could have affected pain scores. However we did not see any reduction in tramadol (reported as morphine equivalents) and morphine dosing in the PACU despite the difference in pain scores. We believe this was due to the PACU nurses treating pain with tramadol or morphine based on clinical judgement and not according to reported pain scores alone.

Depending on the patient, the surgery type and the analgesia regimen, NOL guided anesthesia may lead to an increase or a reduction in intraoperative opioid dosing. In our study there was no difference in the fentanyl dosing between groups. Our results were similar to those achieved by Meijer et al. [11], who showed a decrease in PACU pain scores when fentanyl dosing was guided by the NOL. In summary we attribute the results to more personalized and timely administration of opioids when dosing was guided by the NOL index, as opposed to the usual practice of hemodynamic based dosing. We conclude that inherent inter-patient variability may have a strong effect on opioid dosing requirements and post-operative pain scores. By optimizing the timing of the fentanyl dosing with NOL guidance, patients may have experienced lower levels of sympathetic activation and surgical stress during surgery that translated into reduced PACU pain scores.

## Study limitations

The study protocol did not allow the use of multimodal anesthesia techniques such as TAP (transversus abdominis plane) blocks, epidurals and non-opioid analgesic drugs although these are all broadly used in order to provide more balanced anesthesia and limit the use of opioids, particularly as part of ERAS (Enhanced Recovery After Surgery) and other fast track programs.

The COVID-19 pandemic created challenges in study oversight that resulted in the enrollment of patients that did not fully meet the inclusion criteria. These patients were accordingly excluded from the final analysis. However, as these patients were similarly distributed, we do not believe this had a significant effect on the study results.

As multiple types of surgeries with different durations were included in the study this could have created a confounder affecting the levels of post-operative pain, however this was accounted for in the statistical analysis. In addition, the study was not powered for additional clinical endpoints that may be of interest to hospital administrators such as PACU discharge readiness, post-operative adverse events and the development of chronic pain.

Pain treatment in the PACU was not fully controlled in the study protocol and therefore, tramadol and morphine dosing were based on standard PACU nurse practice according to their clinical judgement and in consultation with the anesthesiologist whilst considering the pain score and the patient’s overall condition. Treatment therefore may not have been strictly driven by the pain scores measured every 15 min. In our hospital, processed EEG depth of anesthesia monitoring is not routinely used and MAC of sevoflurane was used as a target for adequate general anesthesia. However, MAC sevoflurane ranges were similar in both arms as noted.

## Future directions

Larger studies with adequate powering are required in order to study the impact of intraoperative nociception monitoring on perioperative outcome measures. Further studies are required to explore the clinical utility of NOL monitoring when multi modal regimens are implemented. In addition, real world studies would help assess the benefits of nociception monitoring in routine perioperative care in other surgery types.

## Conclusion

Postoperative pain scores were significantly improved in nociception level index-guided patients. We attribute this to more objective and personalized fentanyl dosing based on nociception during anesthesia contributing to lower levels of sympathetic activation and surgical stress.

## Supplementary Information

Below is the link to the electronic supplementary material.

Supplementary file1 (DOCX 14 KB)
