# Supplementary material for: Reduced postoperative pain in patients receiving nociception monitor guided analgesia during elective major abdominal surgery: a randomized, controlled trial
Source: J Clin Monit Comput. 2022 Aug 17;37(2):481–91. doi: 10.1007/s10877-022-00906-1 (PMC9383658; doi:10.1007/s10877-022-00906-1)
Supplement: Supplementary file 1 — Supplementary file1 (DOCX 14 KB) [file 10877_2022_906_MOESM1_ESM.docx]

Cover Letter

July 6, 2022

Editor

Journal of Clinical Monitoring & Computing

Dear Sir,

I am submitting the following article: "Reduced Postoperative Pain in Patients Receiving Nociception Monitor Guided Analgesia During Elective Major Abdominal Surgery: a Randomized Controlled Trial" as a Research Paper for possible publication in the Journal of Clinical Monitoring & Computing.

It was revised according to the reviewers' comments.

The contents of this article have not been published elsewhere and the paper is not being submitted elsewhere.

The manuscript has been read and approved by all co-authors.

Sincerely,

Y. Gozal, MD

Associate Professor of Anesthesiology Hebrew University-Hadassah Medical School

Chair, department of anesthesiology, Perioperative Medicine and Pain treatment Shaare Zedek Medical Center

Jerusalem 9103102, Israel

Tel: 972-2-6555104

Fax: 972-2-6666003

E-mail: [gozaly@szmc.org.il](mailto:gozaly@szmc.org.il)
